# Real-Time Monitoring the Effect of Cytopathic Hypoxia on Retinal Pigment Epithelial Barrier Functionality Using Electric Cell-Substrate Impedance Sensing (ECIS) Biosensor Technology

**DOI:** 10.3390/ijms22094568

**Published:** 2021-04-27

**Authors:** Michael H. Guerra, Thangal Yumnamcha, Abdul-Shukkur Ebrahim, Elizabeth A. Berger, Lalit Pukhrambam Singh, Ahmed S. Ibrahim

**Affiliations:** 1 Department of Ophthalmology, Visual, and Anatomical Sciences, School of Medicine, Wayne State University, 540 East Canfield, Gordon Scott Hall (room 7133), Detroit, MI 48201, USA; michael.guerra2@med.wayne.edu (M.H.G.); gl5948@wayne.edu (T.Y.); ex8029@wayne.edu (A.-S.E.); eberger@med.wayne.edu (E.A.B.); plsingh@med.wayne.edu (L.P.S.); 2 Department of Pharmacology, School of Medicine, Wayne State University, 540 East Canfield, Gordon Scott Hall (room 7133), Detroit, MI 48201, USA; 3 Department of Biochemistry, Faculty of Pharmacy, Mansoura University, Mansoura 35516, Egypt

**Keywords:** age related macular degeneration (AMD), diabetic macular edema (DME), cytopathic hypoxia, retinal pigment epithelial cells (RPE), ARPE-19, CoCl_2_, seahorse, ECIS modeling, R_b_ resistance, alpha resistance, impedance, capacitance, barrier integrity

## Abstract

Disruption of retinal pigment epithelial (RPE barrier integrity is a hallmark feature of various retinal blinding diseases, including diabetic macular edema and age-related macular degeneration, but the underlying causes and pathophysiology are not completely well-defined. One of the most conserved phenomena in biology is the progressive decline in mitochondrial function with aging leading to cytopathic hypoxia, where cells are unable to use oxygen for energy production. Therefore, this study aimed to thoroughly investigate the role of cytopathic hypoxia in compromising the barrier functionality of RPE cells. We used Electric Cell-Substrate Impedance Sensing (ECIS) system to monitor precisely in real time the barrier integrity of RPE cell line (ARPE-19) after treatment with various concentrations of cytopathic hypoxia-inducing agent, Cobalt(II) chloride (CoCl_2_). We further investigated how the resistance across ARPE-19 cells changes across three separate parameters: R_b_ (the electrical resistance between ARPE-19 cells), α (the resistance between the ARPE-19 and its substrate), and C_m_ (the capacitance of the ARPE-19 cell membrane). The viability of the ARPE-19 cells and mitochondrial bioenergetics were quantified with 3-(4,5-dimethylthiazol-2-yl)-2,5-diphenyl-2H-tetrazolium bromide (MTT) assay and seahorse technology, respectively. ECIS measurement showed that CoCl_2_ reduced the total impedance of ARPE-19 cells in a dose dependent manner across all tested frequencies. Specifically, the ECIS program’s modelling demonstrated that CoCl_2_ affected R_b_ as it begins to drastically decrease earlier than α or C_m_, although ARPE-19 cells’ viability was not compromised. Using seahorse technology, all three concentrations of CoCl_2_ significantly impaired basal, maximal, and ATP-linked respirations of ARPE-19 cells but did not affect proton leak and non-mitochondrial bioenergetic. Concordantly, the expression of a major paracellular tight junction protein (ZO-1) was reduced significantly with CoCl_2-_treatment in a dose-dependent manner. Our data demonstrate that the ARPE-19 cells have distinct dielectric properties in response to cytopathic hypoxia in which disruption of barrier integrity between ARPE-19 cells precedes any changes in cells’ viability, cell-substrate contacts, and cell membrane permeability. Such differences can be used in screening of selective agents that improve the assembly of RPE tight junction without compromising other RPE barrier parameters.

## 1. Introduction

Retinal pigment epithelial cells (RPE) constitute a simple layer of cuboidal cells lying in the interface between the outer retina, where the photoreceptors reside, and the choriocapillaris, which forms the outer blood–retinal barrier (oBRB). Tight junctions between adjacent RPE cells are essential in controlling the flow of fluids and molecules that cross the oBRB as well as in preventing the entrance of toxic molecules and plasma components into the retina. When this RPE sealing function is compromised and then the retinal integrity is disrupted, this leads to the progression of various retinal blinding diseases including diabetic retinopathy (DR) and age-related macular degeneration (AMD).

Both DR and AMD impact patients’ vision-related quality of life enormously [1]. In DR, blood content leaking through the compromised RPE barrier into the space between the RPE and the photoreceptors produces diabetic macular edema (DME), the most frequent cause of vision loss among diabetic population [2]. Likewise, as the eyes age, the RPE becomes less able to manage its metabolic load [3] and stressors, such as hypoxia [4] and inflammation [5], leading to RPE cell dysfunction and thus RPE barrier compromise. An impaired RPE barrier allows fluid, solutes, or other factors to accumulate within the retina and disrupt its structure with results ranging from dry AMD to advanced neovascular AMD. The majority of patients have the dry form of AMD which is characterized by the extracellular accumulation of fatty protein deposits called drusen and the degeneration of RPE cells and photoreceptors [6]. The remaining patients suffer from the neovascular form characterized by the abnormal growth of fragile blood vessels from the choroid through compromised RPE layer into the subretinal space, accompanied by hemorrhage of these fragile blood vessels and subsequent blindness (comprehensive reviews on AMD are available elsewhere [7,8,9,10,11]).

Although the cause of disease differs between AMD and DME, the compromise of RPE barrier function plays important roles in both diseases. However, the triggering mechanisms of RPE dysfunction remain incompletely understood. Previous studies have suggested that hypoxia may be a central risk factor for DME [12,13] and AMD [14] as it often precedes the advanced stages of the diseases. Furthermore, it has been reported that mitochondrial function slowly declines with aging [15,16], and is accelerated by diabetes [17], leading to cytopathic hypoxia, where several cell types, including RPE cells, are unable to use oxygen for energy production. Nevertheless, the role of cytopathic hypoxia in RPE barrier dysfunction has not been investigated thoroughly.

Cobalt chloride (CoCl_2_) is widely used as a hypoxia mimetic agent. It has dual effects in inducing cytopathic hypoxia via its competitive inhibitory effect on several iron-dependent proteins involved in oxidative phosphorylation (OxPhos), specifically (Ndufs1 in complex I, Uqcrfs1 in complex III, Cox15 in complex IV, and Atp5 in complex V) and on iron-dependent prolyl hydroxylases involved in controlling hypoxia-inducible factor-α, a transcription factor that acts as a global regulator of oxygen homeostasis [18,19]. Therefore, CoCl_2_-induced cytopathic hypoxia would be a convenient model for investigating the role of cytopathic hypoxia in mediating RPE barrier dysfunction.

Considering how important the RPE maintenance of the outer blood–retina barrier is to the health of the retina, the ability to measure changes to the barrier integrity in real time would be an incredible asset to dynamically quantifying how RPE barrier function changes when exposed to cytopathic hypoxia. The Electrical Cell-substrate Impedance Sensing (ECIS) biosensor technology is a powerful tool for measuring and modelling several parameters related to cellular barrier integrity, as well as for monitoring changes in cell behavior [20]. This is because of two reasons; the first is that ECIS uses a constant alternating current (AC) of 1 µA with a given frequency as a replacement for a direct current (DC). This allows separating the overall impedance (Z; ohms (Ω)) into overall barrier resistance (R, Ω) and cell capacitance (C; farad (F)). R provides measurements related to barrier functions of the cells, whereas C is an indicator for the overall coverage of the cell layer over the substrate. The second reason is related to the multifrequency nature of ECIS, which enables mathematically modeling the R data as a multiparameter contribution of the paracellular junctional space (R_b_), the basolateral adhesion of the cells to the substrate (α), and the capacitance at the cell membrane (C_m_) to the total cell resistance. Thus, the use of ECIS system provides a valuable opportunity to effectively monitor the barrier function of the RPE cells in a noninvasive, continuous manner.

In the present study, we aimed to study the dose-dependent effects of CoCl_2_ on the behavior of ARPE-19 cells. Bioimpedance over a 250− to 64− kHz frequency range was monitored with ECIS Zθ (Applied Biophysics) for ~160 h after cell culturing. Complex impedance measurements, RPE spreading, quality of intercellular junctions, and quality of cell–extracellular matrices adhesions were undertaken to elucidate changes in the observed bioimpedance of RPE cells under the cytopathic hypoxic environment.

## 2. Results

### 2.1. Effects of Cytopathic Hypoxia on ARPE-19 Barrier Function Using Real-Time Bioimpedance Analysis

**Figure 1:**
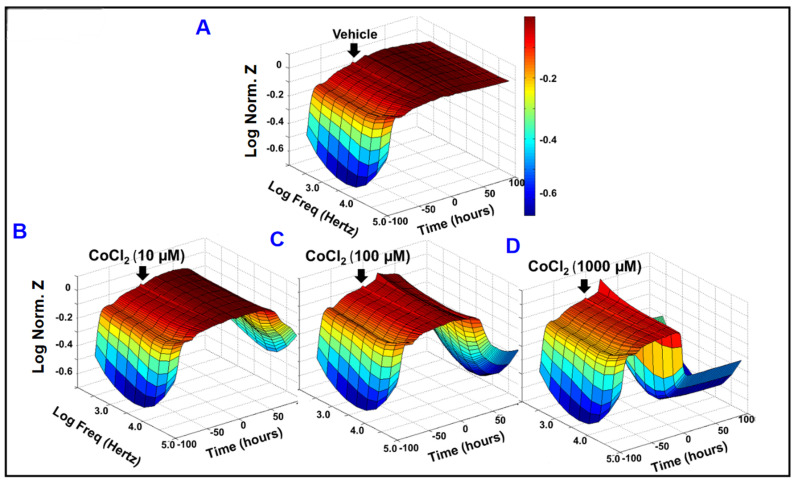
Effects of cytopathic hypoxia on the barrier function of ARPE-19 cells monitored by real-time bioimpedance analysis. Three dimensional representations of the log of normalized impedance across the ARPE-19 cells as a function of both time and of log frequency of the alternating-current (AC) frequency applied to the electrode. T = 0, the time at which control (vehicle) or CoCl_2_ treatment was applied, was 93.17 h after cells were first placed onto the ECIS electrode. Impedance was measured for 100 h after CoCl_2_-treatment at different concentrations (0, 10, 100, and 1000 µM; Figures **A**, **B**, **C**, and **D**, respectively) was applied at T = 0. The value of impedance was normalized to a value of 1 at time T = 0, so all other impedance measurements were calculated as a ratio of Zt/Z0. AC current frequencies used were 250, 500, 1000, 2000, 4000, 8000, 16,000, 32,000, and 64,000 Hz. Abbreviations: Z, impedance; Norm, normalized; Freq, frequency; Zt, impedance at time t; Z0, impedance at time 0.

Given the fact that AMD and DR are associated with impaired barrier function of RPE cells, a functional assay using bioimpedance analysis was carried out in vitro to investigate the role of cytopathic hypoxia in disrupting the barrier function of ARPE-19 cells. The treatment with cytopathic hypoxia-inducing agent (CoCl_2_) at different concentrations (0, 10, 100, 1000 µM; Figure 1A–D, respectively) was initiated after ARPE-19 cells formed mature confluent monolayer as indicated by a plateau in the impedance (Z) on the *y*-axis in the 3D model (represented as log normalized value). Thereafter, the barrier integrity was monitored continuously over a 100 h period (*z*-axis in the 3D model) across frequency range from 250 to 64,000 Hz (represented as log values on the *x*-axis in the 3D model). As shown in Figure 1A–D, CoCl_2_ reduced the total impedance of ARPE-19 cells in a dose dependent manner across all tested frequencies, implicating a role of cytopathic hypoxia in compromising ARPE-19 cell barrier functionality.

**Figure 2:**
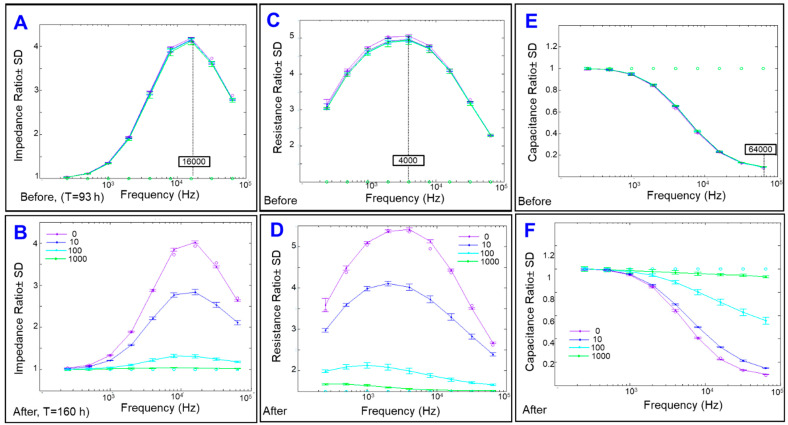
Separation of the impedance into resistance and capacitance. (**A**) Impedance Ratio to the cell-free medium vs. Frequency (Hz) measured at 93 h after ARPE-19 cells placement onto electrodes with a local maximum at 16,000 Hz. (**B**) Impedance Ratio to the cell-free medium vs. Frequency (Hz) measured at 160 h after ARPE-19 cells placement onto electrodes, which is 67 h after application of treatment. (**C**) Resistance Ratio to the cell-free medium vs. Frequency (Hz) measured at 93 h after ARPE-19 cells placement onto electrodes, with a local maximum at 4000 Hz. (**D**) Resistance Ratio to the cell-free medium vs. Frequency (Hz) measured at 160 h after ARPE-19 cells placement onto electrodes. (**E**) Capacitance Ratio vs. Frequency (Hz) measured at 93 h after ARPE-19 cells placement onto electrodes, with a local minimum at 64,000 Hz. (**F**) Capacitance Ratio vs. Frequency (Hz) measured at 160 h after ARPE-19 cells placement onto electrodes.

As the impedance has two components; one due to pure resistance (R) and the other due to capacitance (C), we next aimed to dissect the effect of cytopathic hypoxia on these two components. When cells are challenged with AC, both R and C are created and the Z is the result. However, if cells are challenged with DC, the C disappears and R equals Z. ECIS is an AC measurement which allows monitoring R and C simultaneously [21]. To determine which frequency to use in further evaluations of these parameters, frequency dependence spectra of these parameters were measured. Figure 2A,C,E show the frequency dependence of the Z, R, and C for ARPE-19 cells, respectively, at T = 93 h after placement onto the ECIS sensor and right before initiating any treatment. This is a time at which the cells were confluent, as indicated by a plateau region in the 3D models of Figure 1. The impedance spectrum of ARPE-19 cells showed a characteristic frequency of 16,000 Hz at which maximum Z of ARPE-19 cell occurs, which provides the greatest possible range for group comparison post CoCl_2-_treatment (Figure 2B). On the other hand, looking for the frequency where the resistance is at a maximum, we find that 4000 Hz produces the maximum resistance in ARPE-19 cells. As a result, this frequency was chosen for subsequent analysis of ARPE-19 cells’ barrier properties post CoCl_2_-treatment because it naturally provides the greatest possible range of resistances for studied groups to be compared (Figure 2D).

### 2.2. Real-Time Spreading of ARPE-19 Cells over the Electrode under Cytopathic Hypoxia

**Figure 3:**
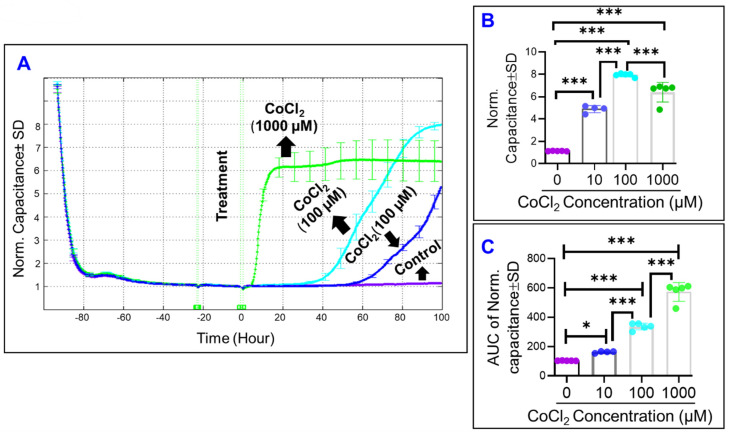
Real-time spreading of ARPE-19 cells over the electrode under cytopathic hypoxia. (**A**) Normalized Capacitance of the ARPE-19 cell groups vs. time, measured at an AC current frequency of 64,000 Hz. Treatments applied at T = 0. Capacitance measured from time of placement onto ECIS electrode to 100 h after treatment application. ARPE-19 cell groups were a control, 10 µM CoCl_2_ treatment, 100 µM CoCl_2_ treatment, and 1000 µM CoCl_2_ treatment groups. (**B**) Bar chart representation of each group’s normalized capacitance at T = 100 h. Statistical comparison analysis performed using ANOVA test followed by Tukey post hoc test. (**C**) Bar chart representation of each group’s area under the normalized capacitance curve for the interval T = 0 to T = 100 h. Abbreviations: AUC, area under the curve; Norm, normalized. Data shown are the mean ± SD of 5 independent biological replicates (*n* = 5/group). *p* values are * *p* ≤ 0.05 and *** *p* ≤ 0.001.

Given the ability of an adherent cell to spread over a substrate has major impact on cell behavior and that there is an inverse relationship between capacitance and cell’s spreading, the spreading of ARPE-19 cells over ECIS electrode was monitored indirectly by measuring the capacitance as a function of time before and after CoCl_2_-treatment (Figure 3A). To do so, we first selected the frequency with which the maximum ARPE-19 cell’s spreading could be detected. As shown in Figure 2E,F, capacitance ratio to the cell-free medium and hence ARPE-19 cell’s spreading are frequency-dependent. We observed that capacitance is minimum at 64,000 Hz, and selected this as the frequency to evaluate ARPE-19 cells’ spreading over the electrode over time because cell’s capacitance is at a minimum when confluence is reached. Figure 3A shows that the capacitance of ARPE-19 cells decreases until it reached a plateau, where CoCl_2_-treatment was given. Thereafter, the capacitance of ARPE-19 cells increased proportionally with increasing concentrations of CoCl_2_. Interestingly, all three concentrations of CoCl_2_ caused significant increases in ARPE-19 cells’ capacitance at the end of the experiment, at T = 100 post-treatment, with no clear dose-related trend (Figure 3B). Instead, a dose-dependent effect of CoCl_2_ on increasing ARPE-19 cells’ capacitance was observed by calculating the area under the curve (AUC), as in Figure 3C. Collectively, these results indicate that ARPE-19 cells change the way they spread over electrodes under cytopathic hypoxia to be less adherent phenotype.

### 2.3. Real-Time Monitoring of ARPE-19 Total Resistance under Cytopathic Hypoxia

**Figure 4:**
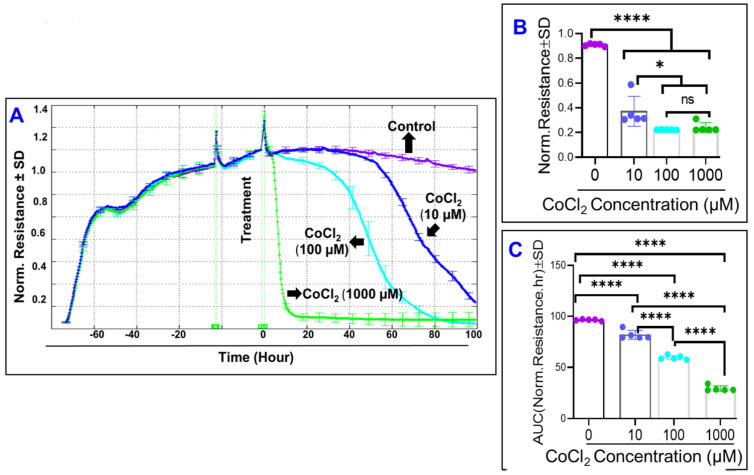
Real-time monitoring of ARPE-19 total resistance under cytopathic hypoxia. (**A**) Normalized Resistance of the ARPE-19 cell groups vs. time, measured at an AC current frequency of 4000 Hz. Treatments applied at T = 0. Resistance measured from time of placement onto ECIS electrode to 100 h after treatment application. ARPE-19 cell groups were a control, 10 µM CoCl_2_ treatment, 100 µM CoCl_2_ treatment, and 1000 µM CoCl_2_ treatment groups. (**B**) Bar chart representation of each group’s normalized resistance at end of experiment T = 100. Using an end-point comparison, the 100 µM group and 1000 µM group display no difference between themselves. (**C**) Bar chart representation of each group’s area under the normalized resistance curve for the interval T = 0 to T = 100 h. Using an area under the curve comparison, each of the 4 groups demonstrates a significant difference compared to each other group. Abbreviations: Norm, normalized; AUC, area under the curve; ns, no significance. Data shown are the mean ± SD of 5 independent biological replicates (*n* = 5/group), *p* values are * *p* ≤ 0.05 and **** *p* ≤ 0.0001.

The total resistance across the ARPE-19 cells was evaluated at 4000 Hz as a function of time over 100 h after treatment. Figure 4A demonstrates that CoCl_2_ induces a dose dependent decrease in the resistance of the ARPE-19 cells, with treatment of 1000 µM CoCl_2_ showing the earliest and greatest deceleration of resistance down to its minimum, whereas the resistance of the 100 µM CoCl_2_ group showing the next change in resistance, and the 10 µM CoCl_2_ group being the last to demonstrate losses in electrical resistance. Not only did each treatment group demonstrate losses to resistance that were significantly different from the control group at the end of the analysis (Figure 4B), but comparisons of area under the curve between each treatment group demonstrated a clear dose-dependent effect, where each treatment group experienced losses to resistance at rates significantly different from each other (Figure 4C).

**Figure 5:**
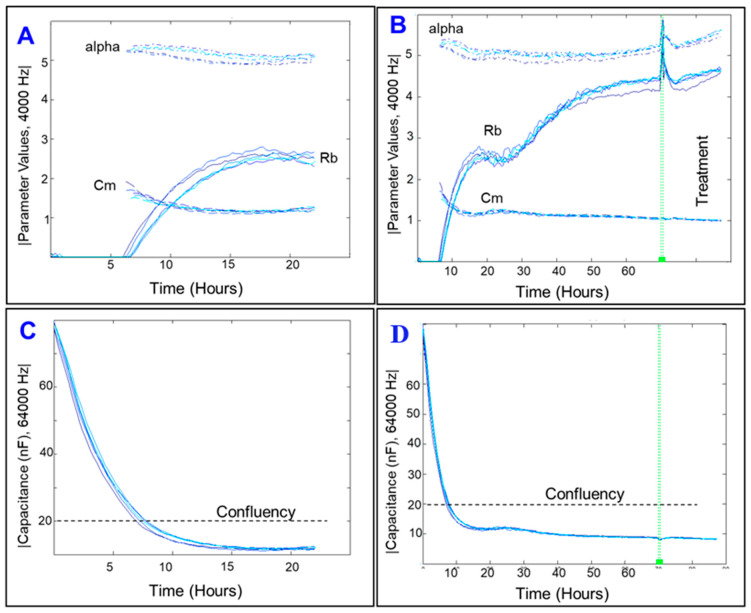
The ECIS models the total resistance across the ARPE-19 cells as three separate components. (**A**,**B**) ARPE-19 cells achieved a stable α resistance as well as Cm and formed a confluent monolayer much before they achieved a stable Rb resistance. The basolateral adhesion of the ARPE-19 cells to the gelatin basement layer (α) was established first and was maximal by ~5 h followed by formation of monolayer by ~8 h as indicated by reaching a plateau in the Capacitance spectrum measured at 64,000 Hz. However, Rb values did not begin to model until ~5 h after the ARPE-19 cells were cultured and reached a maximum at ~60 h later (**C**), where ARPE-19 cells still have a confluent monolayer (**D**). These results indicate that for this specific cell line, a monolayer has formed early, but a functional barrier is not present until ~50 to 60 h after seeding. The green line here represents changing to serum free medium before initiating the treatment. We see in this extended time frame (**C**), that Rb did not maintain the plateau it reached in as seen in (**A**) and instead continues to climb until around T = 60 h. However, α and Cm are steady from T = 20 h until green line. Capacitance is seen to maintain that steady minimum from T = 20 h until green line (**C**,**D**).

**Figure 6:**
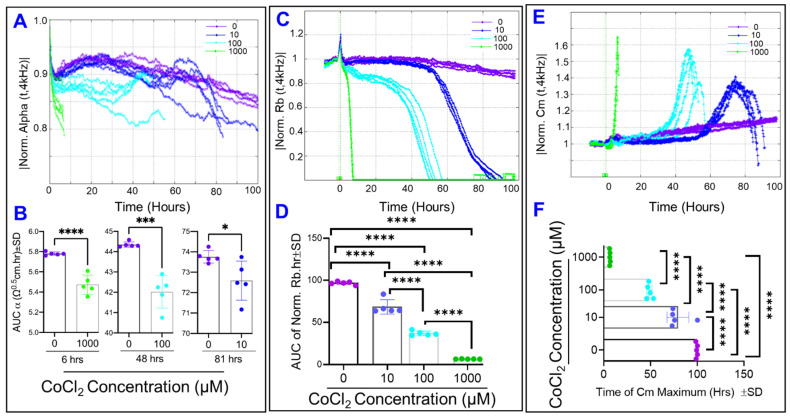
Real-time monitoring of ARPE-19 cells’ α, Rb, and Cm under cytopathic hypoxia. (**A**) Normalized α measured at 4000 Hz vs. time (t) from the time of treatment to T = 100 h after CoCl_2_-treatment at different concentrations (0, 10, 100, and 1000 µM). Considering the curves for the 100 µM and 1000 µM groups, as well as all but one of the curves for the 10 µM group, the curves abruptly end before the 100 h mark is reached. This represents areas where the value could not be modeled as a non-zero or as a positive integer from the total resistance data, as α cannot be calculated when Rb is a non-zero value. The first 10 µM curve to end does so at T = 81 h, the first 100 µM curve to end does so at T = 48 h, and the first 1000 µM curve to end does so at T = 6 h. (**B**) Areas under of normalized α curves. Two tailed t-tests were done to compare treatment group to control, and control is compared to each treatment group at an interval specific to each treatment. The intervals used to calculate the AUCs were as follows: control vs. 10 µM from T = 0−81 h; control vs. 100 µM from T = 0–48 h; and control vs. 1000 µM from T = 0–6 h. These comparisons demonstrate that, in each treatment, α has been reduced by time RB has reached zero. (**C**) Normalized Rb measured at 4000 Hz vs. time (t) from the time of treatment to T = 100 h after CoCl_2_-treatment at different concentrations (0, 10, 100, and 1000 µM). (**D**) Areas under the normalized Rb curve from T = 0–100 h. ANOVA for all groups, demonstrates how Rb also responds to CoCl_2_ with reductions in a dose-dependent fashion. (**E**) Normalized Cm measured at 4000 Hz vs. time (t) from the time of treatment to T = 100 h after CoCl_2_-treatment at different concentrations (0, 10, 100, and 1000 µM). (**F**) Time to maximum value for normalized Cm curve from T = 0–100 h. ANOVA for all groups, demonstrates how C_m_ responds to CoCl_2_ in a dose-dependent fashion. Abbreviations: Norm, normalized; AUC, area under the curve. Data shown are the mean ± SD of 5 independent biological replicates (*n* = 5/group); *p* values are * *p* ≤ 0.05, *** *p* ≤ 0.001, and **** *p* ≤ 0.0001.

Next, these measurements, which represent the overall barrier resistances formed by the ARPE-19 cells consisting of the paracellular barrier resistance (R_b_), the basolateral resistance (α), and the cell membrane capacitance (C_m_), were deconvoluted at 4000 Hz by fitting a mathematical model developed by Giaever and Keese [21] (Figure 5A). As shown in Figure 5, ARPE-19 cells achieved a stable α resistance as well as C_m_ and formed a confluent monolayer much before they achieved a stable R_b_ resistance. The basolateral adhesion of the ARPE-19 cells to the gelatin basement layer was established first and was maximal by ~5 h (Figure 5A) followed by formation of monolayer by ~8 h as indicated by reaching a plateau in the Capacitance spectrum measured at 64,000 Hz (Figure 5B). However, R_b_ values did not begin to model until ~5 h after the ARPE-19 cells were cultured and reached a maximum at ~60 h later (Figure 5A,C), where ARPE-19 cells still have a confluent monolayer (Figure 5D). These results indicate that for this specific cell line, a monolayer has formed early, but a functional barrier is not present until ~50 to 60 h after seeding. This barrier remained relatively stable for the following ~100 h during the window of experiment (Figure 6).

After separating the total resistance into the parameters of α, R_b_, and C_m_, we then measured how each of these parameters changes under cytopathic hypoxia. Figure 6 shows all three parameters are affected by addition of CoCl_2_. CoCl_2_ caused the α resistance to moderately decrease (Figure 6A,B) and the Rb resistance to demonstrably decrease (Figure 6C,D), and it also leads to the C_m_ experience first an increase to a maximum and then decrease thereafter, with increasing dose of CoCl_2_ associated with earlier time when C_m_ acceleration begins (Figure 6E,F). Additionally, when looking at the 10 µM group and comparing when changes are first seen in the two resistance parameters, the paracellular resistance R_b_ appears to be affected first, as it begins to drastically decrease around T = 55 h (Figure 6C), compared to basolateral resistance α which begins to decrease compared to control around T = 80 h (Figure 6A). The cell membrane capacitance C_m_ appears to increase around the same T = 55 h mark as the R_b_ begins its descent (Figure 6E).

**Figure 7:**
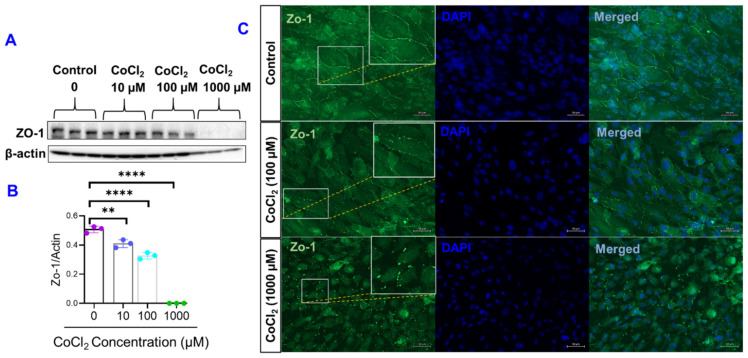
Effects of cytopathic hypoxia on zonula occludens (ZO)-1 expression in ARPE-19 cells. (**A**) Western blot analysis of ZO-1 (198 kD); β-actin (43 kD) was used as the loading control. (**B**) Quantification of densitometric scans of protein bands showing significant decreases in ZO-1 expression in the ARPE-19 cells treated with different concentrations of CoCl_2_ for 60 h versus controls. Data shown are the mean ± SD of 3 independent biological replicates (*n* = 3/group). *p* values are ** *p* ≤ 0.01 and **** *p* ≤ 0.0001. (**C**) Representative photographs of ZO-1 immunofluorescence (green) in ARPE-19 cells treated with different concentrations of CoCl_2_ versus vehicle-treated controls. Enlarged pictures were shown in squares pointing to the punctate pattern alteration in ZO-1 distribution at cellular border of CoCl_2_ -treated ARPE-19 cells; scale bar = 100 µm; and the blue staining (DAPI) is a nuclear marker.

The finding that CoCl_2_ compromised the ARPE-19 paracellular barrier resistance (measured by ECIS) in a dose dependent manner has been further substantiated by testing different concentrations of CoCl_2_ on modulating the expression of zonula occludens (ZO)-1, a tight junction protein that governs the paracellular permeability of endothelial and epithelial cells. Concordantly, Western blot and densitometric analysis of ZO-1 revealed that CoCl_2_ induced a dose-dependent decrease of ZO-1 protein expression in ARPE-19 cells (Figure 7A,B). To further verify the effect of CoCl_2_ on ZO-1 expression pattern, ARPE-19 cells were treated with CoCl_2_ and examined for ZO-1 by immunofluorescence. Consistently, smooth and continuous staining for ZO-1 along the intercellular borders of ARPE-19 cells was seen in controls as shown in Figure 7C. However, treating ARPE-19 with CoCl_2_ (in particular 100 and 1000 µM) caused a punctate pattern alteration in ZO-1 distribution at cellular border, displaying the discontinuity of tight junctions (Figure 7C).

### 2.4. Effects of Cytopathic Hypoxia on ARPE-19 Cell Viability

**Figure 8:**
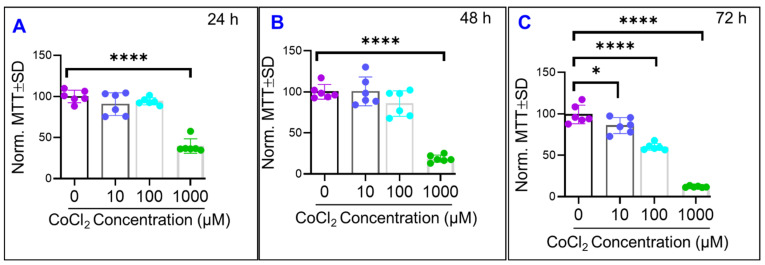
Effects of cytopathic hypoxia on ARPE-19 cell viability. MTT assays were performed on our control and treatment groups at 3 discrete time points in the experiment. (**A**) MTT response of each group at 24 h after treatment application. Only the 1000 µM of CoCl_2_ and the control group are different from each other at this time. (**B**) MTT response at 48 h after treatment, again where only the 1000 µM CoCl_2_ group and control are different from each other. (**C**) MTT response at 72 h post treatment, where each treatment group now demonstrates a significant difference when compared to control group. Data shown are the mean ± SD of 6 independent biological replicates (*n* = 6/group); *p* values are * *p* ≤ 0.05 and **** *p* ≤ 0.0001.

After showing that CoCl_2_ dose-dependently disrupted the barrier integrity of ARPE-19 cells, our interest in understanding the underlying mechanisms was expanded to study whether this effect is a consequence of affecting ARPE-19 cell’s viability or not. To this end, we performed MTT assay for each CoCl_2_ concentration at three time points. The MTT assay results in Figure 8 show that at both 24 and 48 h after CoCl_2-_treatment, only the 1000 µM treatment group displays decreased viability compared to control, and then at 72 h all three CoCl_2_ treatment groups show decreased viability compared to control. However, in looking at the 100 µM CoCl_2_ group in both Figure 4A and C, by both hour 24 and hour 48 the 100 µM CoCl_2_ group has demonstrated decreases to both its total resistance and paracellular resistance when compared to control. Conversely, at each of these time points the MTT assay did not demonstrate any loss in viability of the 100 µM CoCl_2_ group yet, suggesting that the disruption of barrier integrity of ARPE-19 cells in response to cytopathic hypoxia is an earlier event occurs before any noticeable effect on cell viability.

### 2.5. Effects of Cytopathic Hypoxia on ARPE-19 Cell Mitochondrial Bioenergetics

**Figure 9:**
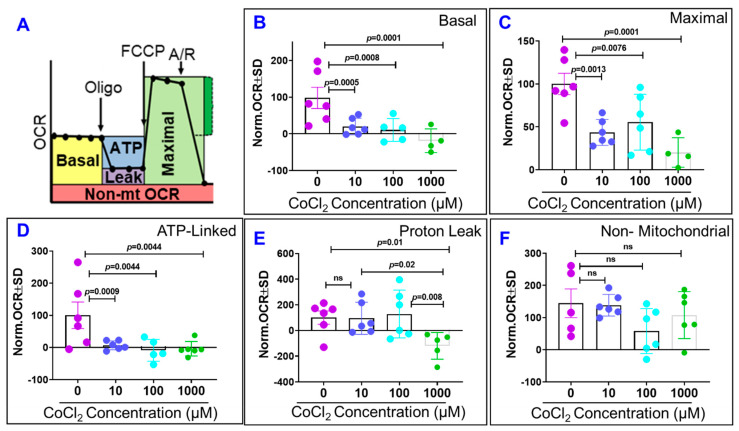
Effects of cytopathic hypoxia on ARPE-19 cell mitochondrial bioenergetics. ARPE-19 cells were incubated with different concentrations of CoCl_2_ (0, 10, 100, 1000 µM) for 48 h before measuring oxygen consumption rate (OCR) with XFp Cell Mito Stress kit (**A**). The optimal concentrations of oligomycin (Oligo), FCCP, and antimycin/rotenone (A/R) were titrated (1 μM/each), data not shown. We found that all three concentrations of CoCl_2_ after 48 h significantly impaired basal, maximal, and ATP-linked respirations of ARPE-19 cells (**B**–**D**, respectively), but they did not affect proton leak and non-mitochondrial bioenergetic (**E**,**F**, respectively). Data are normalized means of OCR (pmol/minute) ± SD to control and analyzed by Tukey’s post hoc test. Data shown are the mean ± SD of 5–6 independent biological replicates (*n* = 5–6/group); 30,000 cells of ARPE-19/well. Ns; non-significant.

To determine whether the negative effect of CoCl_2_ on ARPE-19 cell barrier function is associated with reduced mitochondrial bioenergetics, we used XF^e^96 Seahorse analyzer to monitor oxygen consumption rate (OCR, an indicator of mitochondrial respiration) in living ARPE-19 cells. Using Mito Stress Test, whereby sequential addition of respiratory chain inhibitors and activators was carried out as depicted in Figure 9A, we were able to dissect which mitochondrial bioenergetic function was affected by cytopathic hypoxia. Initially, we measured baseline OCR, from which basal respiration was derived by subtracting non-mitochondrial OCR. Next, we added oligomycin (Oligo, 1 µM, a complex V inhibitor), and we used the resulting OCR to measure both ATP-linked respiration (by subtracting the Oligo rate from basal OCR) and proton leak respiration (by subtracting non-mitochondrial OCR from the Oligo rate). Next, we added (Tri-fluoromethoxy carbonylcyanide phenylhydrazone (FCCP), 1 µM) to collapse the inner membrane gradient, allowing the mitochondrial respiration to function at its maximal rate, where maximal respiration was calculated by subtracting non-mitochondrial rate from the FCCP rate). Lastly, we added rotenone/antimycinA (1 µM/each) to shut down mitochondrial respiration by inhibiting complex I and III, respectively, and to calculate the non-mitochondrial OCR. We found that all three concentrations of CoCl_2_ after 48 h significantly impaired basal, maximal, and ATP-linked respirations of ARPE-19 cells (Figure 9B–D, respectively), but they did not affect proton leak and non-mitochondrial bioenergetic (Figure 9E).

## 3. Materials and Methods

### 3.1. Human Retinal Pigmented Epithelial Cell Line (ARPE-19)

ARPE-19 cells obtained from American Type Culture Collection (ATCC; CRL-2302) were grown in Dulbecco’s modified Eagle’s medium-nutrient mixture F-12 (DMEM/F-12, Corning-10-090-CV) supplemented with 10% Fetal bovine serum (FBS, Corning-35011CV) and 1% penicillin/streptomycin (PS; Hyclone- SV30010). Before treatment with different concentrations of CoCl_2_ (Sigma; 15862-1ML-F), ARPE-19 cells were shifted to the serum free media for 4–8 h.

### 3.2. Conducting ECIS Experiment and Modelling

Effects of CoCl_2_ on barrier function of ARPE-19 were evaluated by monitoring changes in overall impedance (Z; ohms (Ω)). Normalized Z was recorded by Electric Cell-substrate Impedance Sensing (ECIS^®^Zθ (theta)) biosensor technology (Applied Biophysics Inc) as previously described [22]. Briefly, a 96-wells arrays (96W20idf PET, Applied Biophysics Inc) were coated with 50 µL of 100 µM cysteine for 30 min followed by coating with 50 µL of 0.02% gelatin (Sigma; G1393) for 30 min. Thereafter, ARPE-19 cells were seeded in DMEM/F12 full media containing 10% FBS and 1% PS. After ARPE-19 cells reached the confluency, indicated by a capacitance below 20 nF, they were serum starved then treated with different concentrations of CoCl_2_. The ECIS system continually measured the total electrical impedance across the mono-layer of cells, and impedance was measured with respect to both time and to the frequency of the 1 µA AC current applied to the electrode. Multifrequency measurements of 250, 500, 1000, 2000, 4000, 8000, 16,000, 32,000, and 64,000 Hz were taken for each of the 96 wells at a fixed 180 s interval. Impedance for each well was normalized by dividing the measured impedance at each time point by the baseline impedance acquired before the addition of the treatment and then plotted as a function of time. The ECIS technology has the capability of calculating both the resistance and capacitance of the cell monolayer as a function of the total impedance measured across the ARPE-19 cells. The ECIS technology is also able to separate this total resistance into three distinct parameters: R_b_ (the electrical resistance between ARPE-19 cells, ohms-cm^2^), α (the basolateral resistance between the ARPE-19 and its substrate, ohms-cm^1/2^), and C_m_ (the capacitance of the ARPE-19 cell membrane, µF/cm^2^). R_b_, the resistance between ARPE-19 cells, represents the integrity of the paracellular junctions. α represents the integrity of the ARPE-19 cells’ basolateral attachment to its substrate. C_m_ is considered the same for both the basolateral and apical membrane as the current model is unable to distinguish the two. After collection of this electrical impedance data, we used the ECIS data collection and analysis software to determine total resistance. Resistance for each well was normalized by dividing the measured resistance at each time point by the baseline resistance measured in that well just before the addition of the treatment at time T = 0; this normalized resistance was plotted as a function of time. Then the ECIS software used this resistance data to model the three parameters of the total resistance. The areas under the normalized curves of resistance were chosen as the comparison measurement to compare how the treatments differed across the entire 100 h of the experiment, instead of at the end point or a collection of middle points. Using comparison of the areas under the normalized curves also allows for combined comparison of differences in both rates of change as well as absolute values over time.

### 3.3. Immunofluorescence of Zonula Occludens (ZO)-1

ARPE-19 cells were stained with zonula occludens (ZO)-1 antibody according to our previous procedure [22]. Briefly, ARPE-19 cells were fixed in paraformaldehyde (4%, 10 min) followed by one-hour blockage in 0.2% gelatin in 0.3% tritonX-PBS. Next, ARPE-19 cells were incubated with anti-ZO-1 (Invitrogen, Catalog # 40-2200 1:100) overnight at 4 °C followed by an incubation with VectaFluor™ Horse Anti-Rabbit IgG, DyLigh 488 labeled secondary antibody (Vector Laboratories, Catalog#DI-1788). Images were taken by Apotome microscope Zeiss with 20X objective lens (Carl Zeiss, Thornwood, NY, USA).

### 3.4. Assessment of Cytopathic Hypoxia Toxicity with MTT

The effect of various concentrations of CoCl_2_ on ARPE-19 cells’ viability was performed with 3-(4,5-dimethylthiazol-2-yl)-2,5-diphenyltetrazolium bromide (MTT; Invitrogen- M6494) assay. ARPE-19 Cells were grown in 96-well plates (1 × 10^4^/200 μL/well). After incubation with CoCl_2_ (0, 10, 100, and 1000 μM), the medium was removed and the cells were treated with 20 μL of MTT (5 mg/mL) for 3 h at 37 °C. Subsequently, 100 μL of DMSO (Sigma; D2650) were added. Then, the solubilized formazan product was spectrophotometrically quantified by a microplate reader (Synergy HI Hybrid Reader, BioTek) at 540 nm.

### 3.5. Western Blot Analysis

ARPE-19 cellular lysates were prepared for analyzing ZO-1 protein expression by Western blot analysis. Briefly, ARPE-19 cells were resuspended in RIPA lysis buffer containing protease and phosphatase inhibitors as previously described [23]. ZO-1 protein expression was detected by anti-ZO-1 antibody (Invitrogen, Catalog # 40-2200 1:1000) followed by a horseradish peroxidase-conjugated antibody and enhanced chemiluminescence detection system (Thermo Fisher Scientific, Rockford, IL, USA). Blot images were taken by Azure Biosystem C500 (Dublin, CA, USA) and protein expression was quantified by Image Studio Lite software after normalizing to β-actin.

### 3.6. Measurement of Mitochondrial Bioenergetics

XFe96 flux bioanalyzer (Seahorse Bioscience) was used to monitor mitochondrial bioenergetic by measuring oxygen consumption rate (OCR) as described before [24,25]. Briefly, one hour prior to OCR measurements, the culture media of ARPE-19 cells (40,000 cells/well) was replaced by XF assay media. This was followed by sequential injection of selected mitochondrial inhibitors included in the mito-stress test kit (Agilent; 103010-100); oligomycin (Olig, 1 µM), FCCP (1 µM), and rotenone/Antimycin (1 µM each), to determine precisely which parameters of five main mitochondrial functions was affected by different concentrations of CoCl_2_: basal OCR, ATP-linked OCR, proton leak OCR, maximal OCR, and non-mitochondrial OCR.

### 3.7. Statistical Analysis

Differences between experimental groups were assessed by the two-tailed *t* test or one-way analysis of variance (ANOVA) followed by Tukey test. Graphical representations of *p* values are * *p* ≤ 0.05, ** *p* ≤ 0.01, *** *p* ≤ 0.001, **** *p* ≤ 0.0001. Experiments were repeated at least in two different batches of cells. Only one set of experiments is presented in the figures. The number of biological replicates is included within the figure legends.

## 4. Discussion

The key finding of our study is that cytopathic hypoxia differentially disrupts the barrier integrity of ARPE-19 cells across three distinct domains: the barrier between cells, the barrier between the cells and their basolateral substrate, and the barrier to flow through the cells. More specifically, the paracellular junctions are the most vulnerable target for cytopathic hypoxia. The following evidence supports this conclusion: (a) the R_b_ component of the ARPE-19 barrier was the parameter affected earliest and greatest by cytopathic hypoxia in a dose dependent fashion; (b) other components of the ARPE-19 barrier including flow beneath the ARPE-19 cells (α) or charge flow through the cells (C_m_) were not compromised at the time when R_b_ started to decline; and (c) interestingly, this breakdown effect of cytopathic hypoxia on paracellular junctions was not a consequence of ARPE-19 cell death. Our study is the first to show these temporal relationships between ARPE-19 barrier parameters in response to cytopathic hypoxia using ECIS mathematical modeling system.

It has been validated that changes occurring in R_b_ measurements were correlated with changes in the expression of key junctional proteins, including the transmembrane proteins occludins and claudins, and the cytoplasmic scaffolding proteins zonula occludens (ZO), which are known to provide paracellular barrier strength [20]. Furthermore, the ZO-1 belongs to a superfamily of proteins known as the membrane-associated guanylate kinases (MAGUK), and all members of this family share conserved tyrosine residues. As such, ZO-1 is considered as a target for tyrosine kinase activators, in particular VEGF receptor-2 activation in epithelium [26]. Thus, it is suggested that phosphorylation of ZO-1 by hypoxia-induced VEGF secretion is involved in the regulation of paracellular permeability [27].

It is worth mentioning that the ARPE-19 cells prioritize formation of their basolateral substrate attachments long before they establish and strengthen their paracellular tight junctions (Figure 5). However, under stress condition of cytopathic hypoxia, the ARPE-19 cells showed a weakening of their basolateral attachment, as seen in Figure 6A,B where the α value is reduced from control before the R_b_ value reaches zero. As the ECIS is unable to model values for α when the R_b_ value reaches zero, we cannot determine from this data whether there is a dose dependent effect of CoCl_2_ on the strength of the ARPE-19 basolateral attachments. Additionally, we see that in cytopathic hypoxia, the reduction in strength of the basal adhesion are much less intense than those losses to the paracellular attachments and thus the tight junctions, again seen in Figure 6A,C. Thus, we can infer that in a state of hypoxia losses of the total barrier created by the ARPE-19 cells is largely due to loss of tight junctions, as the R_b_ value is what reaches zero far before the model is even able to calculate values of α near zero. Studies of endothelial cells of the blood brain barrier have also exemplified this trend of cells types that maintain barriers and tight regulation of flow. In a study where endothelial cells of the blood brain barrier were put in a minimal media environment upon the ECIS electrode, the cell experienced significant losses to their total resistance and R_b_ with minimal losses in α, reflecting the primary importance of the paracellular tight junctions to the total barrier functionality [20].

The basolateral attachment of the ARPE-19 cells to its substrate is mediated by RPE surface integrins bound to the extracellular matrix components like laminin, fibronectin, vitronectin, and collagen [28,29]. In vivo, the RPE are adhered to the extracellular proteins of Bruch’s membrane on their basolateral surface. Increasing the activity of these RPE integrins has been shown to enhance attachment of the RPE cells to a Bruch’s membrane that has been affected by neo vascular AMD [30]. On the other hand, oxidative stress has been shown to disrupt RPE adhesion to the ECM [31]. Considering how the production of ROS is increased in conditions of hypoxia due to disruptions in mitochondrial electron transport [32], the oxidative stress experienced by the RPE cells during cytopathic hypoxia is a potential contributing factor to the reduced basolateral attachment elucidated in our experiment. Another avenue to explain the decrease in the basolateral barrier is that hypoxic induction of apoptosis drives basolateral detachment. We first look at our 100 µM CoCl_2_ group and notice in Figure 8B at 48 h the cells were still viable, yet Figure 6C shows that the paracellular barrier and tight junctions have been clearly compromised by hour 48. Additionally, Figure 6A shows in the α curve that by 48 h the 100 µM CoCl_2_ group experienced no clear loss in in basolateral adhesion. Furthermore, looking at our 100 µM CoCl_2_ treatment group and comparing Figure 6A,C and Figure 8C, we see that by 72 h the paracellular barrier is significantly weakened and the cells demonstrate loss of viability, but the α value is just beginning to decline if not still steady at this time until then dropping later around hour 80. The third explanation for loss of basolateral adhesion under cytopathic hypoxic is due to activation of caspases, which then leads to a cascade including inactivation of focal adhesion kinase, whose inhibition disrupts normal integrin signaling and results in loss of basal adhesion [33]. Likely maintenance of a strong connection to the RPE substrate is so important to cell survival that significant losses to the basolateral barrier occur primarily after cell viability is compromised.

Following this further, our experiment demonstrated that the RPE cells exhibit an increase to membrane capacitance early in response to cytopathic hypoxia and after enough time this membrane capacitance C_m_ drops, as seen in Figure 6E. For all three groups this increase in membrane capacitance begins around the same time that R_b_ begins to steeply decelerate as shown in Figure 6C. However, membrane capacitance falls after it reaches a maximum and then never recovers or changes course while Rb is still a decelerating, non-zero value. One reason for this behavior of the C_m_ is an evolution to the cellular geometry during this cytopathic hypoxia. C_m_ has been shown to vary directly with cell surface area. Early in hypoxia, the decreased production of ATP reduces activity of cell surface sodium–potassium pumps [34], leading to cellular swelling and thus an increase in cell surface area, which would be reflected by an increase in membrane capacitance. When the cells then reach cell death during this cytopathic hypoxia, the cell surface area shrinks as the RPE cells undergo autophagy in their process of programmed cell death [35]. Another explanation to the changes in membrane capacitance is related to changes in membrane permeability. Flow of current and molecules through the RPE is largely mediated by ion pumps and channels found on the RPE membrane [36]. A rising capacitance reflects an increase in the electric charge that is held at the inner surface of the cell membrane, and thus in our experiment it may represent increased flow of electric charges into the cell through the transcellular barrier to flow, that being the cell plasma membrane. One method of transcellular flow involves membrane transporters; potentially in hypoxic conditions either expression or activity of membrane channels and transporters is increased. RPE cells have been shown to express acid sensing ion channels (ASICs) [37], which are cation channels whose activity increase in states where extracellular pH decreases. As the extracellular fluid of our RPE is becoming more acidic while the cells perform anaerobic glycolysis and lactic acid production, increased activity of these ASICs potentially contributes to the increased permeability to charge flow at the RPE membrane. Efflux and influx transporters have been found to be increased in expression in RPE during states of hypoxia [38], and as such their increased activity during hypoxia represents another potential avenue of transcellular flow. In fact, photoreceptors require high amount of glucose for its bioenergetics and phototransducing activity. The byproduct lactate is secreted into the interphotoreceptor matrix (IPM) between the RPE and photoreceptor, which is taken up by RPE for its energy production through oxidative phosphorylation [39]. The fact that RPE cells under cytopathic hypoxia are utilizing glucose by anaerobic glycolysis implies that photoreceptor energy deficiency may occur under various age-related retinal neurodegenerative diseases. Additionally, the expression of water transporters on the RPE membrane in the form of aquaporins has also been shown to be enhanced in a state of cytopathic hypoxia [40], which is consistent with our findings that the transcellular flow of material through the RPE is elevated under hypoxic conditions. Furthermore, studies have shown that in a state of hypoxia, cell membranes experience lipid peroxidation and membrane damage [41,42], and as the plasma membrane is a vital barrier to the transcellular flow of material, this peroxidation and membrane damage likely increases the flow of charge through the RPE cells in our experiment.

Considering how the barrier function of the RPE is known to be weakened in pathologic states of vision loss such as AMD and DME, future studies can make use of the ECIS system to determine how these conditions affect the components RPE barrier function. Additionally, studies aimed at evaluating and manipulating gene expression of RPE cells subjected to these pathologic states will be useful to finding gene targets related to RPE paracellular barrier degeneration. Conceivably, future studies can also utilize the ECIS to measure the transepithelial and paracellular resistance of RPE cells harvested from AMD or DME patients in order to profile the resistances of the pathologic cells. Exposing those cells to treatments during resistance measurement can be done to find the interventions that best ameliorate loss of the RPE barrier function over time, or possibly even mend this barrier after damage has occurred. The tight regulation of the barrier between the retina and the choroidal blood supply is imperative to retinal health, and the ECIS will be a powerful tool to elucidate how chronic insults to the RPE disrupt the components of this barrier and then to discover how to recover its integrity.

Collectively, our data demonstrate that the ARPE-19 cells have distinct dielectric properties in response to cytopathic hypoxia in which disruption of barrier integrity between ARPE-19 cells precedes any changes in cells’ viability, cell-substrate contacts, and cell membrane permeability. However, the significance of these findings in other independent RPE sources, such as iPSC RPE or primary RPE, needs to be further examined.
